# *β*‐caryophyllene and *β*‐caryophyllene oxide—natural compounds of anticancer and analgesic properties

**DOI:** 10.1002/cam4.816

**Published:** 2016-09-30

**Authors:** Klaudyna Fidyt, Anna Fiedorowicz, Leon Strządała, Antoni Szumny

**Affiliations:** ^1^ Laboratory of Tumor Molecular ImmunobiologyLudwik Hirszfeld Institute of Immunology and Experimental Therapy, Polish Academy of Sciences12 Rudolf WeiglWroclaw53‐114Poland; ^2^ The Faculty of Food ScienceDepartment of ChemistryWrocław University of Environmental and Life Sciences25/27 C.K. NorwidaWroclaw50‐375Poland

**Keywords:** Analgesic, anticancer, antinociception, cannabinoid receptor type 2 (CB_2_), *β*‐caryophyllene (BCP), *β*‐caryophyllene oxide (BCPO)

## Abstract

Natural bicyclic sesquiterpenes, *β*‐caryophyllene (BCP) and *β*‐caryophyllene oxide (BCPO), are present in a large number of plants worldwide. Both BCP and BCPO (BCP(O)) possess significant anticancer activities, affecting growth and proliferation of numerous cancer cells. Nevertheless, their antineoplastic effects have hardly been investigated in vivo. In addition, both compounds potentiate the classical drug efficacy by augmenting their concentrations inside the cells. The mechanisms underlying the anticancer activities of these sesquiterpenes are poorly described. BCP is a phytocannabinoid with strong affinity to cannabinoid receptor type 2 (CB_2_), but not cannabinoid receptor type 1 (CB_1_). In opposite, BCP oxidation derivative, BCPO, does not exhibit CB_1/2_ binding, thus the mechanism of its action is not related to endocannabinoid system (ECS) machinery. It is known that BCPO alters several key pathways for cancer development, such as mitogen‐activated protein kinase (MAPK), PI3K/AKT/mTOR/S6K1 and STAT3 pathways. In addition, treatment with this compound reduces the expression of procancer genes/proteins, while increases the levels of those with proapoptotic properties. The selective activation of CB_2_ may be considered a novel strategy in pain treatment, devoid of psychoactive side effects associated with CB_1_ stimulation. Thus, BCP as selective CB_2_ activator may be taken into account as potential natural analgesic drug. Moreover, due to the fact that chronic pain is often an element of cancer disease, the double activity of BCP, anticancer and analgesic, as well as its beneficial influence on the efficacy of classical chemotherapeutics, is particularly valuable in oncology. This review is focused on anticancer and analgesic activities of BCP and BCPO, the mechanisms of their actions, and potential therapeutic utility.

## Introduction

**Figure 1:**
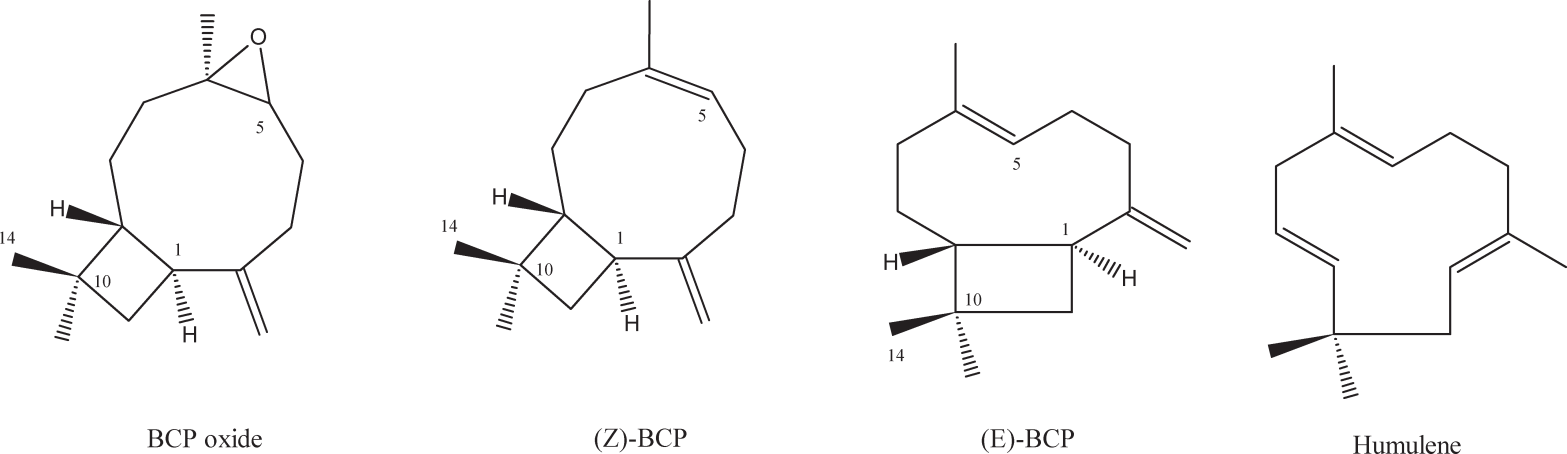
Trans‐caryophyllene, its isomers, and oxidative product.

*β*‐caryophyllene (BCP) is a plant compound, a member of bicyclic sesquiterpene. In nature, it mainly occurs as trans‐caryophyllene ((E)‐BCP) mixed with small amounts of its isomers, (Z)‐*β‐*caryophyllene (iso‐caryophyllene) and *α*‐humulene (*α*‐caryophyllene), as well as its oxidation derivative—*β*‐caryophyllene oxide (BCPO) (Fig. 1). In this review, we will focus on two sesquiterpenes, BCP (in the scientific literature, BCP mainly stands for (E)‐BCP or the natural mixture of BCP isomers) and BCPO.

BCP and BCPO have strong wooden odor and they are used as cosmetic and food additives. These two natural substances are approved as flavorings by the Food and Drug Administration (FDA) and by the European Food Safety Authority (EFSA) with identification number FL no: 01.007 for BCP and FL no: 16.043 for BCPO. Both compounds exhibit low water solubility, thereby the aqueous medium such as biological fluids, impede their absorption to the cell. However, it was shown that both BCP and BCPO are able to interact with artificial lipid bilayer, which strongly suggests their high affinity to the cell membrane 1. The potential obstacles associated with poor solubility of these sesquiterpenes in aqueous fluids may be overcome through usage of liposomal drug delivery system, which provides much higher bioavailability of these compounds and by that ensures obtaining desired biological effects.

BCP is one of the major active component of essential oils derived from large number of spice and food plants. According to Essential Oil Database (EssOilDB) (http://nipgr.res.in/Essoildb/), BCP as a plant volatile compound is commonly found in basil (*Ocimum* spp.), cinnamon (*Cinnamomum* spp.), black pepper (*Piper nigrum* L.), cloves (*Syzygium aromaticum*), cannabis (*Cannabis sativa* L.), lavender (*Lavandula angustifolia*), oregano (*Origanum vulgare* L.), and rosemary (*Rosmarinus officinalis*). Its biological effects include anti‐inflammatory 2, anticarcinogenic 3, antimicrobial 4, antioxidative 5, and analgesic activities 6.

Similarly to BCP, BCPO due to its high biological activity was extensively studied in recent years. EssOilDB‐based data indicate basil (*Ocimum* spp.), salvia (*Salvia glutinosa*) and Syzygium cordatum as the main natural sources of BCPO. Either as a pure substance or a component of plant essential oils, BCPO was found to exhibit anti‐inflammatory 7, antioxidant, antiviral 8, anticarcinogenic 9, and analgesic properties 10.

**Figure 2:**
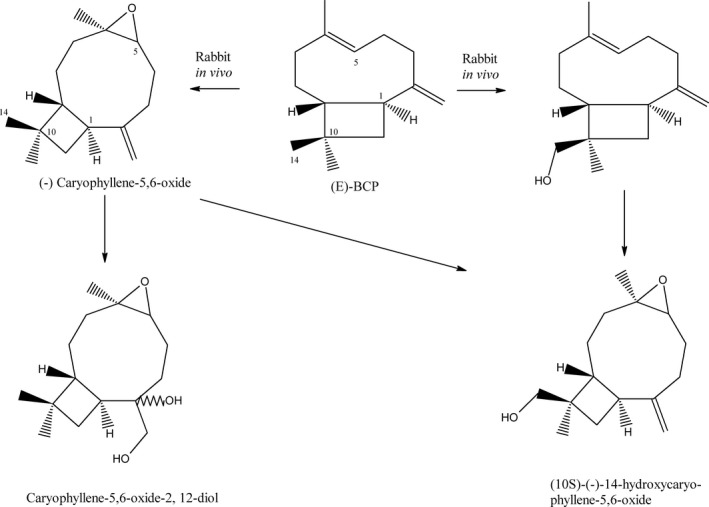
The metabolism of (E)‐BCP in rabbits. Based on Asakawa et al. (1986). BCP, *β*‐caryophyllene.

The metabolism of BCP(O) is poorly described. While BCP metabolic pathway was investigated in rabbits, there is some information on BCPO biotransformation. In vivo tests performed on rabbits revealed that (E)‐BCP is converted to intermediate metabolite, (–)‐caryophyllene‐5,6‐oxide, which is metabolized to [10S‐(−)‐14‐hydroxycaryophyllene‐5,6‐oxide] or hydroxylated to by‐product, caryophyllene‐5,6‐oxide‐2,12‐diol (Fig. 2) 11. By comparison with rabbit metabolic pathway, one can suspect that BCP may undergo sequential transformations also in humans, however the experimental data confirming this hypothesis is lacking 11. Interestingly, Hart and Wong 12 evaluated BCP toxicity in rats and found that oral lethal dose (LD_50_) for this compound was higher than 5000 mg/kg.

BCP belongs to a class of cannabinoids (CBs), specifically phytocannabinoids (pCBs), which were identified as plant derivatives of *Cannabis sativa* L. Natural and synthetic cannabinoids have ability to activate the cannabinoid receptors (CB_1_ and CB_2_), however BCP, which is common in essential oil from *C. sativa* (up to 37%) 13, activates exclusively CB_2_ and exhibits no affinity to CB_1_. This implies that BPC action is devoid of psychoactive side effects associated with CB_1_ activation and suggests its potential use in medicine. The quantitative radioligand‐binding experiments showed that E‐BCP displays insensibly higher biding affinity to CB_2_ than its isomer Z‐BCP, whereas BCPO and *α*‐humulene possess no CB_2_ binding properties. In addition, all these compounds did not bind to CB_1_ 14. Lack of affinity of BCPO to CB_2_ clearly shows that both chemically related compounds, BPC and BCPO, exert their biological activities though at least partially different mechanisms.

## Cannabinoid Receptors

Cannabinoid receptors—cannabinoid receptor type 1 (CB_1_) and type 2 (CB_2_)—are G‐protein‐coupled receptors (GPCR) and main components of endocannabinoid system (ECS). They play important roles not only in the maintenance of energy balance, metabolism, neurotransmission, and immune response, but are also engaged in pathological processes, for example, neuropathic pain 15, 16, 17. CB_1_ and CB_2_ differ essentially in their structures, ligands, cellular distributions, and topologies. CB_1_ are mostly localized to the central nervous system (CNS), whereas CB_2_ are found predominantly in the peripheral tissues and immune cells. However, immunohistochemical studies revealed that CB_2_ are also expressed in the brain, glial cells, and neurons 18, 19. Both types of CB receptors are elements of numerous signaling pathways, mediating cellular responses to various bioactive molecules such as hormones, local mediators, or neurotransmitters. For that reason, they are also involved in pathomechanisms of many clinical conditions such as obesity, osteoporosis, neurodegenerative/neuroinflammatory disorders, psychiatric diseases, stroke, and spinal cord injury 20, 21, 22.

BCP binding to CB_2_ results in the activation of G_*α*i/o_ protein, which leads to decline of cAMP production and in consequence inhibition of adenylyl cyclase. In addition, ligand‐coupled CB_2_ activate G*γβ* proteins and stimulate both mitogen‐activated protein kinase (MAPK) and phosphoinositide 3‐kinase (PI3K) signaling pathways 23. Moreover, the chemical modifications of BCP have impact on its activity through generating molecules with different affinities to CB_1/2_, thus altered pharmacological traits 24.

## BCP(O) as Anticancer Agents

Many investigations have been made to establish the potential utility of cannabinoids in cancer therapies. Currently, it is believed that all anticancer activities of cannabinoids may be based on three different mechanisms such as (1) induction of apoptosis 25, (2) repression of cell cycle 26, and (3) inhibition of angiogenesis and metastasis 27. The anticancer properties of BCP and BCPO are less recognized than those of traditional cannabinoids, however several lines of evidence have demonstrated that these natural compounds can be interesting candidates for complementary treatment of the cancer. Both sesquiterpenes revealed cytotoxic activities against several types of cancer cells. It was shown that BCPO isolated from Jeju guava (*Psidium cattleianum* Sabine) leaves exerted cytotoxic effect on various cancer cell lines, such as HeLa (human cervical adenocarcinoma cells), HepG2 (human leukemia cancer cells), AGS (human lung cancer cells), SNU‐1 (human gastric cancer cells), and SNU‐16 (human stomach cancer cells). Interestingly, comparative data analysis has shown that dose of BCPO and time required for BCPO‐induced cytotoxicity was specific for each studied cell line 28. Moreover, Shahwar et al. 29 noted that BCPO derived from *Cinnamomum tamala* leaf extracts exhibited moderate cytotoxic activity against human ovarian cancer cell line, A‐2780. The antiproliferative effect of BCP on several cancer cell lines was reported by Dahham et al. 30. They found that treatment with BCP obtained from essential oils of *Aquilaria crassna* stem bark led to strong growth inhibition in two colon cancer cell lines, HCT‐116 and HT‐29, as well as in pancreatic cancer cells, PANC‐1, whereas other tested cancer cell lines demonstrated moderate susceptibility to BCP. In contrast, Ambrož et al. 31 studies revealed that BCP isolated from *Myrica rubra* did not affect CaCo‐2 intestinal cancer cell viability at used doses. On the other hand, BCP isomer, *α*‐humulene, exhibited significant antiproliferative activities against those cells. Moreover, the cytotoxic effect of not only *α*‐humulene, but also iso‐caryophyllene, was enhanced by BCP. Furthermore, both isomers combined with BCP were more effective in reduction of MCF‐7 human breast cancer cell line proliferation than when used separately 32. Amiel et al. 33 demonstrated that treatment of BS‐24‐1 (mouse lymphoma cell line—T cells) and MoFir (human B lymphocytes transformed with Epstein–Barr virus) cells with BCP‐activated caspase‐3 and led to internucleosomal fragmentation of DNA, one of the main features of apoptosis. Analogous changes were observed by Dahham et al. 30 in HCT‐116 cells treated with BCP derived from the essential oil of *A. crassna*. Interestingly, Amiel et al. 33 showed that human skin fibroblast (FB) were resistant to *Commiphora gileadensis* stem extracts, in which BCP was a major compound.

Despite many reports on antiproliferative and cytotoxic properties of BCP(O) toward numerous cancer cell lines, there is only limited data supporting the antitumor efficacy of these compounds in animal models. Jung et al. 34 described in their excellent work the effects of BCP treatment on the multiple cancer parameters in obese mice. Authors observed that animals fed the high‐fat diet (HFD) and injected with B16F10 melanoma cells were prone to form larger and more aggressive tumors than their lean counterparts, and BCP treatment abolished the HFD procancer effects. The anticancer activity of BCP in vivo was also presented at the Euro Global Summit on Cancer Therapy in Valencia, 2015 35. In this report, a growth and vascularization of tumors developed from orthotopically grafted colon cancer cells into nude mice were reduced significantly after administration of BCP isolated from agar wood. Interestingly, Campos et al. 36 demonstrated an additional bioactivity of BCP, which could be useful in cancer therapy. Thus, they found that BCP treatment alleviated the leukopenia induced by the experimental chemotherapy in rats. Taking into account the strong evidence of BCP(O) antineoplastic actions in vitro, there is an urgent need to test these compounds in animal model systems. This is particularly important since up to now only one peer‐reviewed report describing an in vivo effect of BCP on tumor growth exists in the scientific literature. Moreover, there is some information on BCPO antitumor activity in animal models.

Aside from the direct anticancer activities, BCP and BCPO have ability to enhance the efficacy of classical anticancer drugs, such as paclitaxel or doxorubicin (DOX) 31, 32, 37. Ambrož et al. 31 have reported that BCPO potentiated the anticancer activities of DOX toward CaCo‐2 cells. Authors noted that cotreatment with BCPO increased the concentration of DOX in CaCo‐2 cells in dose‐dependent manner leading ultimately to accumulation of the drug in the cells. Likewise, BCPO was shown to improve the anticancer effectiveness of paclitaxel 37, which is a microtubule toxin with ability to arrest cells in mitosis by interfering with normal breakdown of microtubules during cell division 38. Kim et al. 37 found a potentiating influence of BCPO on DOX and paclitaxel anticancer activities in human myeloid leukemia (KBM‐5), multiple myeloma (U266), and human prostate cancer (DU145) cell lines. Furthermore, Legault et Pichette 32 showed that BCP can also increase the anticancer drug efficacy. Thus, they observed the enhancement of paclitaxel activity in MCF‐7 (breast cancer), DLD‐1 (colon cancer), and L‐929 (murine fibroblast) cells cotreated with BCP. Interestingly, in DLD‐1 cell line, BCP induced the accumulation of paclitaxel inside the cells 32, thus exhibited the analogs mechanism of action to that of BCPO. The ability of BCP to increase the intracellular concentrations of anticancer drugs may be linked to its chemical structure of sesquiterpene. Namely, various cyclic hydrocarbons such as terpenes may assemble in the cell membrane leading to higher bilayer permeability 39. Thus, it is likely that BCP is incorporated into the membrane of cancer cell, making it more available for entering the drugs.

## The Mechanisms of BCP(O) Anticancer Activities

Many experiments have been performed in order to elucidate the mechanisms of anticancer activities of BCPO. On the contrary, the mechanisms underlying the antineoplastic actions of BCP have hardly been studied. It seems that among these two compounds, BCPO possesses stronger anticancer properties, which can be explained by its chemical structure. Thus, BCPO contains methylene and epoxide exocyclic functional groups, therefore it binds covalently to proteins and DNA bases by sulfhydryl and amino groups. For that reason, BCPO reveals high potential for being signaling modulator in tumor cancer cells 40. Anticancer activities of both sesquiterpenes may be exerted through suppression of cellular growth and induction of apoptosis. Park et al. 40 showed that BCPO suppressed PC‐3—prostate cancer cell and MCF‐7—breast cancer cell proliferation in a dose‐dependent manner. Moreover, it induced ROS generation, MAPK activation, and inhibition of PI3K/AKT/mTOR/S6K1 signaling pathway in these cells, a pathway which is essential in cell survival, proliferation, and angiogenesis of the tumor 41. Furthermore, the authors found that BCPO significantly reduced levels of procancer proteins, those involved in proliferation—cyclin D1, metastasis—COX‐2 (cyclooxygenase 2), angiogenesis—VEGF (vascular endothelial growth factor), and apoptosis inhibitors—bcl‐2 (B‐cell lymphoma 2), bcl‐xL (B‐cell lymphoma extra‐large), IAP‐1, IAP‐2 (inhibitor of apoptosis 1 and 2), and survivin. In contrast, a treatment with this natural compound augmented the expression of tumor suppressors—p53 and p21—in PC‐3 cells 40. Suppression of AKT/mTOR/S6K1 signaling in PC‐3 cells was also reported after treatment with hexane fraction obtained from guava leaf (*Psidium guajava* L.), in which BCPO was a major bioactive constituent 42. BCPO also targets STAT3 (Signal Transducer and Activator of Transcription 3) signaling pathway, which is involved in proliferation, survival, invasion, angiogenesis, and metastasis of cancer and was found to be highly active in many human tumors 43. Kim et al. 44 observed the reduced activity of STAT‐3 transcription factor after BCPO treatment in multiple melanoma, breast, and prostate cancer cell lines. They reported that suppression of STAT3 pathway by BCPO was mediated through activation of SHP‐1 protein tyrosine phosphatase. Moreover, BCPO was capable to block the IL‐6‐induced activation of STAT‐3 and the upstream elements of STAT3 pathway, such as c‐Src, JAK1, and JAK2, in time‐ and dose‐dependent manners.

Proapoptotic activity of BCPO in cancer cells can be associated with reduced activation of NF‐*κ*B 37. NF‐*κ*B is one of the key transcription factors in tumor development, controlling such processes as cancer cell proliferation, tumorigenesis, angiogenesis, and metastasis 45. NF‐*κ*B regulates expression of a large number of genes, involved in cellular proliferation, apoptosis, and inflammation (e.g., TRAF—TNF receptor‐associated factor, c‐FLIP—cellular FLICE‐like inhibitory protein, survivin, various chemokines, and cytokines). Kim et al. 37 reported BCPO‐induced inhibition of the constitutive and inducible NF‐*κ*B activities in cancer cells. Moreover, they found that BCPO increased the TNF*α*‐caused apoptosis by inhibiting the NF‐*κ*B activation. In addition, treatment with BCPO led to lowering the levels of cyclin D1, COX‐2, and c‐Myc, which expression was upregulated by TNF*α*. Sain et al. 46 evaluated an influence of BCP and BCPO fractions from *Aegle marmelos* extract on IMR‐32 human neuroblastoma and Jurkat cell lines. They found that treatment of the cells with these chemical fractions led to induction of p53‐dependent apoptosis. Cellular death was accompanied by upregulation of proapoptotic gene expression, namely those encoding p53, bax, bak1, caspase 8, caspase 9, and ATM as well as decrement of mRNA levels of antiapoptotic genes, such as bcl‐2, mdm2, COX‐2, and c‐myb.

Taking together, BCP(O) present the anticancer activities toward numerous cancer cell lines, however strength of the cellular response induced by treatment with these compounds differs substantially among cancer cells. Doses used in in vitro studies described in this review are listed in Table 1. Moreover, the antitumor potential of BCP(O) still needs to be evaluated in in vivo systems. Interestingly, BCP(O) has ability to potentiate the efficacy of classical drugs by augmenting their concentrations inside the cells. The mechanisms underlying the antineoplastic effects evoked by these sesquiterpenes are poorly recognized. One can assume that BCP exerts its action through binding to CB_2_. In contrast, BCPO does not display any affinity to CB_1/2_, but reveals the equally strong (or ever stronger) anticancer activity than BCP. It is known that BCPO alters several key pathways for cancer development, such as MAPK, PI3K/AKT/mTOR/S6K1, and STAT3 pathways. In addition, treatment with this compound reduces the expression of procancer genes/proteins, while increases levels of those with proapoptotic properties.

**Table 1:** Concentrations of BCPO and BCP used in in vitro studies of BCP(O) anticancer activities

	Concentration (*μ*g/mL)	Cell line	Author
BCPO			
Isolated from *Psidium cattleianum* Sabine IC_50_	0.87	HepG2	Jun et al. 28
	2.98	HeLa	
	2.77	AGS	
	3.69	SNU‐1	
	6.03	SNU‐16	
Isolated from *Cinnamomum tamala* leaves extract IC_50_	8.94	A‐2780	Shahwar et al. 29
	7.19	BHK‐21	
Purchased from Sigma‐Aldrich IC_50_	57.7	CaCo‐2	Ambrož et al. 31
Purchased from Jeju National University, Korea	6.6	KBM‐5, H1299, A293, U266, DU145	Kim et al. 37
Purchased from Jeju National University, Korea	6.6	PC‐3, MCF‐7	Park et al. 40
Purchased from Jeju National University, Korea	2.2	DU145, MDAMB‐231	Kim et al. 44
	6.6	U266, MM1.S	
BCP			
Isolated from essential oils of *Aquilaria crassna* IC_50_	3.9	HCT 116	Dahham et al. 30
	5.5	PANC‐1	
	12.9	HT‐29	
	19.4	ME‐180	
	21.3	PC3	
	21.5	K562	
	58.2	MCF‐7	
IC_50_ (source unknown)	64 lack of anticancer effects	DLD‐1/L‐929	Legault, Pichette^32^
Purchased from Sigma‐Aldrich	4.9 × 10^−5^	BS‐24‐1, MoFir	Amiel et al. 33

BCP(O) concentrations are shown as: IC_50_, half maximal inhibitory concentration or the lowest concentration used exhibiting antiproliferative/cytotoxic activity. BCPO, *β*‐caryophyllene oxide; BCP, *β*‐caryophyllene.

## BCP(O) as Analgesic Agents

Pain is a subjective sensation, evoked by various internal and external stimuli. In biological aspect, it is unpleasant feeling, which arises from sensitization of nociceptors—peripheral neurons responding to pain stimuli. Acute but in particular chronic pain is a serious social burden, it affects quality of life and leads to economic loss for patients as well as health services 47. It has been estimated that around 10% of population worldwide suffers from long‐lasting pain 48.

One of the most difficult pain to manage is cancer related. Many factors may be involved in etiology of cancer pain, such as progression/invasion of the tumor, surgical procedures and other cancer treatments, cancer‐related infections, etc. 49, which makes it complicated to treat. As a consequence, a large part of oncological patients tend to overuse the synthetic or semisynthetic pain killers such as opioids or nonsteroidal anti‐inflammatory drugs (NSAIDs). Prolonged consumption of these medicines may cause serious side effects leading to health complications as well as drug tolerance and addiction. In order to decrease a use of synthetic drugs, the natural products with strong analgesic activities and low side effects are still being sought. On account of that, cannabinoid receptors have been extensively studied as mediators of analgesia and thus potential targets for treatment of acute and neuropathic pain 50. Activation of those receptors by endo‐ and exogenous ligands may inhibit pain responses, therefore CBs are considered as substances with high analgesic activities. One of the best studied natural product, which contains large amount of cannabinoids, is cannabis, also known as marijuana. Medicinal marijuana with THC (tetrahydrocannabinol) as a major constituent is approved for the supportive care of several medical conditions in Austria, Belgium, Canada, and several states of the United States 51.

BCP is a selective agonist of CB_2_, which is predominantly expressed on the periphery. Thereby pain modulation by BCP could be largely mediated through non‐neuronal cells. In contrast to anticancer research, most of the studies on analgesia focus on BCP, since BCPO does not bind to CB_2_. However, there is some evidence that BCPO can exert its antinociceptive action beyond cannabinoid system machinery.

For reliable evaluation of BCP analgesic properties, all data described in this review were obtained with use of animal models of acute or chronic pain. Kuwahata et al. 52 employed the mouse models of neuropathic pain to assess whether BCP evokes antinociception through activation of CB_2_ or CB_1_. In these experiments, the animals were administered with CB_2_ and CB_1_ antagonists, AM630 and AM251, respectively, before BCP injection. The results have shown an inhibition of analgesic effects of BCP by pretreatment with AM630, but not with AM251, which proved that antiallodynic actions of BCP are exerted only through activation of local peripheral CB_2_. Analgesic efficacy of oral BCP treatment in mouse models of inflammatory and neuropathic pain was investigated by Klauke et al. 6. The antinociceptive properties of BCP were evaluated on wild‐type, CB_2_(^+/+^), and knockout, CB_2_(^−/−^), mice. Similarly to studies of Kuwahata et al. 52, BCP acted as an analgesic agent by activation of CB_2_ since antipain effect of BCP was not observed in CB_2_(^−/−^) animals. Interestingly, BCP can diminish an acute and chronic pain not only through cannabinoid, but additionally through opioid system. This was observed in mice after oral administration of BCP, in which licking and jumping latency in the hot plate test was increased, whereas pain feeling in the formalin test was attenuated 53. In contrast to BCP, BCPO does not attract much attention as a pain modulator, although it may possess some antinociceptive properties since Chavan et al. 54 have documented centrally and peripherally mediated analgesia by BCPO isolated from *Annona squamosa* bark extract, in response to pain stimuli in mice.

Interestingly, pure BCP displays similar analgesic activities as several essential oils, in which BCP is a major active compound. Thus, oils extracted from *Dracocephalum kotschyi* 55, *Hyptis fruticosa* 56, *Teucrium stocksianum* 57, *Peperomia serpens* 58, *Vitex agnus‐castus* 59, and *Hyptis pectinata* 60 alleviated pain sensation to similar extent as BCP, which was shown in rodent pain models such as writhing 55, 56, 57, 58, 59, formalin 58, 59, 60, hot plate 56, and tail immersion 59 tests. However, it should be noted that essential oils are mixture of various chemical compounds, which may potentially modulate the antinociceptive action of BCP.

One can hypothesize that better analgesic effects may be obtained when BCP is used in combination with other natural agent(s) of desired properties. For this purpose, Fiorenzani et al. 61 studied the antinociceptive activity of BCP in mixture with docosahexaenoic acid (DHA). DHA is a member of omega‐3 polyunsaturated fatty acids (PUFAs) and well‐known anti‐inflammatory mediator 62. Thus, a combination of BCP and DHA was suspected to bring a double, analgesic and anti‐inflammatory effect in the treatment of inflammation‐associated pain. However, it turned out that mixture of BCP+DHA did not exert an additional analgesic activity over that of BCP alone in animal model of formalin‐induced pain. On the other hand, the same study has revealed that DHA attenuated BCP toxicity in fibroblasts.

To understand better the BCP‐mediated analgesia, it is essential to get insight into mechanism of its action. It still requires elucidation, however current knowledge about this compound allows for some assumptions to be made. As phytocannabinoid, it may act in a similar manner to other CB_2_‐selective agonists. CB_2_ activation can mediate antinociception either directly or indirectly, where direct activity is exerted through CB_2_ stimulation on primary sensory neurons 63. In contrast, indirect analgesic responses are related to inhibition of the release of proinflammatory factors or/and may engage other systems involved in analgesia, such as endogenous opioid system 64. The literature data indicate that CB_2_‐selective agonists stimulate peripheral release of endogenous opioids such as *β*‐endorphins, which activates *μ*‐opioid receptors on primary afferent neurons 65. In inflammatory hyperalgesia, indirect pain inhibition through CB_2_ localized on mast and immune cells is possibly achieved by the reduction of prostanoids or cytokines release, which are responsible for peripheral nociceptor sensitization. Other CB_2_‐dependent analgesic activities, which are not associated with inflammation, such as inhibition of nerve injury‐induced sensory hypersensitivity or inhibition of acute thermal nociception, are still indeterminate 66. Fernandes et al. 67 found that BCP derived from essential oil of *Cordia verbenacea* exhibited anti‐inflammatory properties, blocking release of proinflammatory molecules, such as TNF*α* and prostaglandin E2 (PGE2). The same report showed BCP‐induced decrement in expression of COX‐2 and inducible nitric oxide synthase (iNOS), which could suppress the NF‐*κ*B activation and in a consequence promote analgesia. In addition, Paula‐Freire et al. 53 reported a decreased level of IL‐1*β* in the injured sciatic nerve after BCP treatment, in a model of chronic pain. Another possible mechanism of BCP pain modulation may be related to peripheral CB_2_ simulation and *β*‐endorphin release from keratinocytes, which was noted after local and intraplantar injections of BCP in response to capsaicin‐induced nociception. Interestingly, Katsuyama et al. 68 showed that BCP potentiated an analgesic action of morphine, thereby combination therapy with BCP may be suggested in order to reduce doses and common side effects of this opioid agent.

## Conclusions

**Figure 3:**
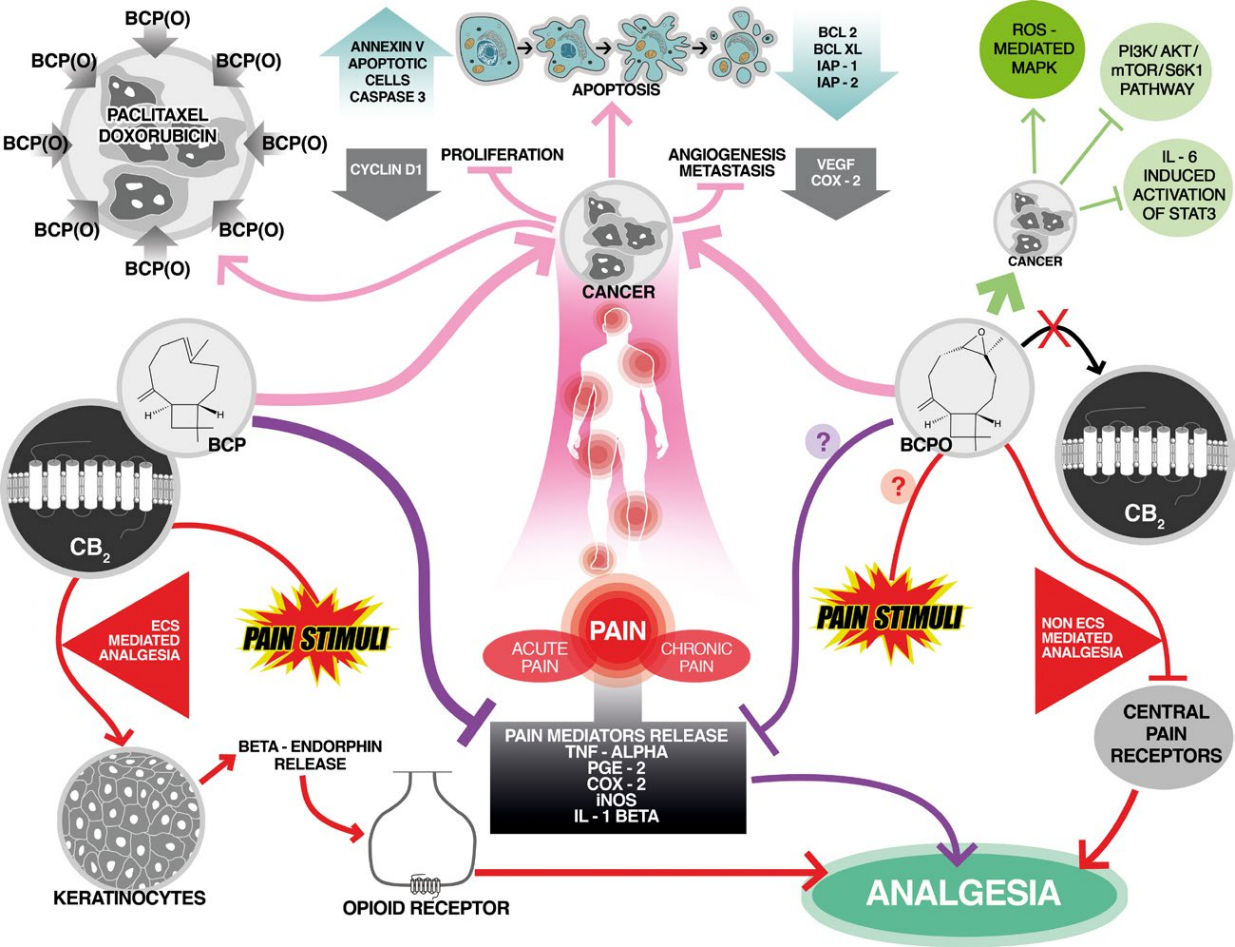
Anticancer and analgesic activities of *β*‐caryophyllene (BCP) and *β*‐caryophyllene oxide (BCPO). BCP and BCPO induce apoptosis and suppress proliferation of cancer cells as well as reduce levels of tumor angiogenesis and metastasis markers. Molecular mechanisms of BCPO anticancer activities include activation of mitogen‐activated protein kinase (MAPK) pathway as well as inhibition of PI3K/AKT/mTOR/S6K1 and STAT3 signaling. Additionally, BCP(O) increase cellular accumulation of chemotherapeutic drugs, enhancing their anticancer effectiveness. In response to pain stimuli, BCP and BCPO reveal different mode of actions. BCP‐induced effect of analgesia is obtained with endocannabinoid system (ECS) involvement, while BCPO analgesic activity is ECS independent. BCP binds to peripheral cannabinoid receptor type 2 (CB_2_) leading to *β*‐endorphin release from keratinocytes and activation of opioid receptors. In contrast, antipain effects of BCPO are possibly achieved by inhibition of central pain receptors. Additionally, both compounds inhibit the release of inflammatory mediators of pain.

We have presented in this review that natural products, BCP(O), have strong potential for being used in medical applications, due to their anticancer and analgesic properties (Fig. 3). Both compounds could be applied in alternative therapy of cancer, supporting the conventional forms of treatment. Since BCP(O) enhances the efficacy of some chemotherapeutics, they could be employed in combination therapy with the classical anticancer drugs. BCP has also the ability to reduce pain, without causing psychoactive side effects, as other CB_1_ agonists do, which makes it particularly valuable in chronic pain treatment. Moreover, BCP and BCPO could be used in a mixture as they often occur in plants. In a medical practice, the application of such BCP/BCPO mixture in combination with the classical anticancer drugs could bring many benefits, thus could potentiate the efficacy of used chemotherapeutics, elicit the supplementary antineoplastic effect, as well as reduce the refractory cancer pain at the same time. However, this potential triple activity of BCP/BCPO need to be carefully evaluated in animal models of cancer and cancer pain. Importantly, BCP and BCPO are found in reasonable amounts in wide range of plants and are well tolerated at high doses, thus easily accessible and safe. Despite the fact that both sesquiterpenes can be potentially useful in medicine, the metabolic, biochemical, and molecular characteristics of these natural compounds are still humble and need further investigations.

## Conflict of Interest

None declared.
